# Labial Cartilaginous Choristoma Mimicking a Mucocele: A Rare Case Report and Literature Review

**DOI:** 10.1002/ccr3.73396

**Published:** 2026-08-26

**Authors:** Kimia Hafezimotlagh, Saede Atarbashi‐Moghadam, Ali Lotfi, Bahram Ardalani, Mohammadreza Kashefi Baher

**Affiliations:** ^1^ Department of Oral Medicine, School of Dentistry Tehran University of Medical Sciences Tehran Iran; ^2^ Department of Oral and Maxillofacial Pathology, School of Dentistry Shahid Beheshti University of Medical Sciences Tehran Iran; ^3^ Health Research Center, Chamran Hospital Tehran Iran

**Keywords:** cartilage, choristoma, lip, oral cavity

## Abstract

Choristomas are non‐neoplastic, tumor‐like lesions composed of histologically normal tissue in ectopic locations, though their exact pathogenesis remains unknown. Labial cartilaginous choristoma is an exceptionally rare benign lesion that clinically mimics common lip swellings, posing a diagnostic challenge. A 25‐year‐old male presented with a painless, slow‐growing submucosal swelling of the right lower labial mucosa, measuring 1 cm in diameter, that had been present for 2 years. Clinical examination revealed a firm, mobile, pink nodule with an intact overlying mucosa and focal hyperkeratosis, without associated trauma or relevant medical history. The differential diagnoses included mucocele and focal fibrous hyperplasia. Following excisional biopsy, histopathologic examination with hematoxylin and eosin staining demonstrated a well‐defined lesion composed of mature cartilaginous tissue admixed with fibrous and adipose elements, devoid of cytological atypia, confirming the diagnosis of cartilaginous choristoma. Complete surgical excision was diagnostic and associated with an excellent clinical outcome. A review of the literature identified 12 previously reported cases of labial choristoma, with the cartilaginous type predominating (53.85%), a slight female predilection (F:M ratio 1.17), and most presenting as asymptomatic swellings. No recurrence has been documented following adequate excision at the labial site. This case highlights that labial cartilaginous choristoma, though rare, should be considered in the differential diagnosis of persistent lip swellings, as definitive diagnosis relies on histopathologic evaluation, and complete surgical excision appears curative.

## Key Clinical Message

Labial cartilaginous choristoma is a rare benign lesion that may clinically mimic mucoceles or fibromas. Histopathologic examination remains essential because clinical features alone cannot reliably distinguish this lesion from common benign lip swellings. Awareness of this entity helps avoid misdiagnosis and unnecessary treatment delays.

## 1 Introduction

Choristomas are non‐neoplastic, tumor‐like lesions consisting of histologically normal tissue located in ectopic sites, although their exact pathogenesis remains unclear [1, 2]. These lesions can be classified based on their histological components. While cartilaginous and osseous types are the most common, choristomas may also include neuroglial tissue, respiratory or gastrointestinal mucosa, thyroid tissue, fat, and muscle [1, 3]. The term “cartilaginous choristoma” is preferred over the traditionally and inaccurately used designation “soft tissue chondroma”, as it underscores the non‐neoplastic nature of the lesion [4, 5].

Oral choristomas show a higher prevalence in females; however, their age distribution remains unclear, as cases have been reported across all age groups [1, 6]. Oral cartilaginous choristomas are rare lesions, most frequently affecting the tongue. Involvement of other intraoral sites, such as the buccal mucosa, gingiva, palate, and lip, has been reported but is considerably less common [7, 8]. The histopathologic classification and anatomical location of the lesion may influence the patient's presenting chief complaint. In this context, Shareef et al. [1] reported that more than half of patients with osseous lingual choristoma presented with gagging/globus sensation, dysphagia, or hoarse speech. Moreover, labial cartilaginous choristomas are generally asymptomatic [2, 6, 8]; nevertheless, painful cases of labial osseous choristomas have also been documented [9, 10].

The central rationale for standardized reporting of the present case of cartilaginous choristoma of the lower lip lies in its clinical overlap with common lip nodules (e.g., mucocele and fibroma), as well as the rarity of this lesion, which has resulted in limited demographic, clinical, and histopathologic data. Moreover, the accompanying literature review was conducted to broaden current knowledge on labial choristomas and help address the existing gaps in the literature.

## 2 Case History/Examination

**FIGURE 1:**
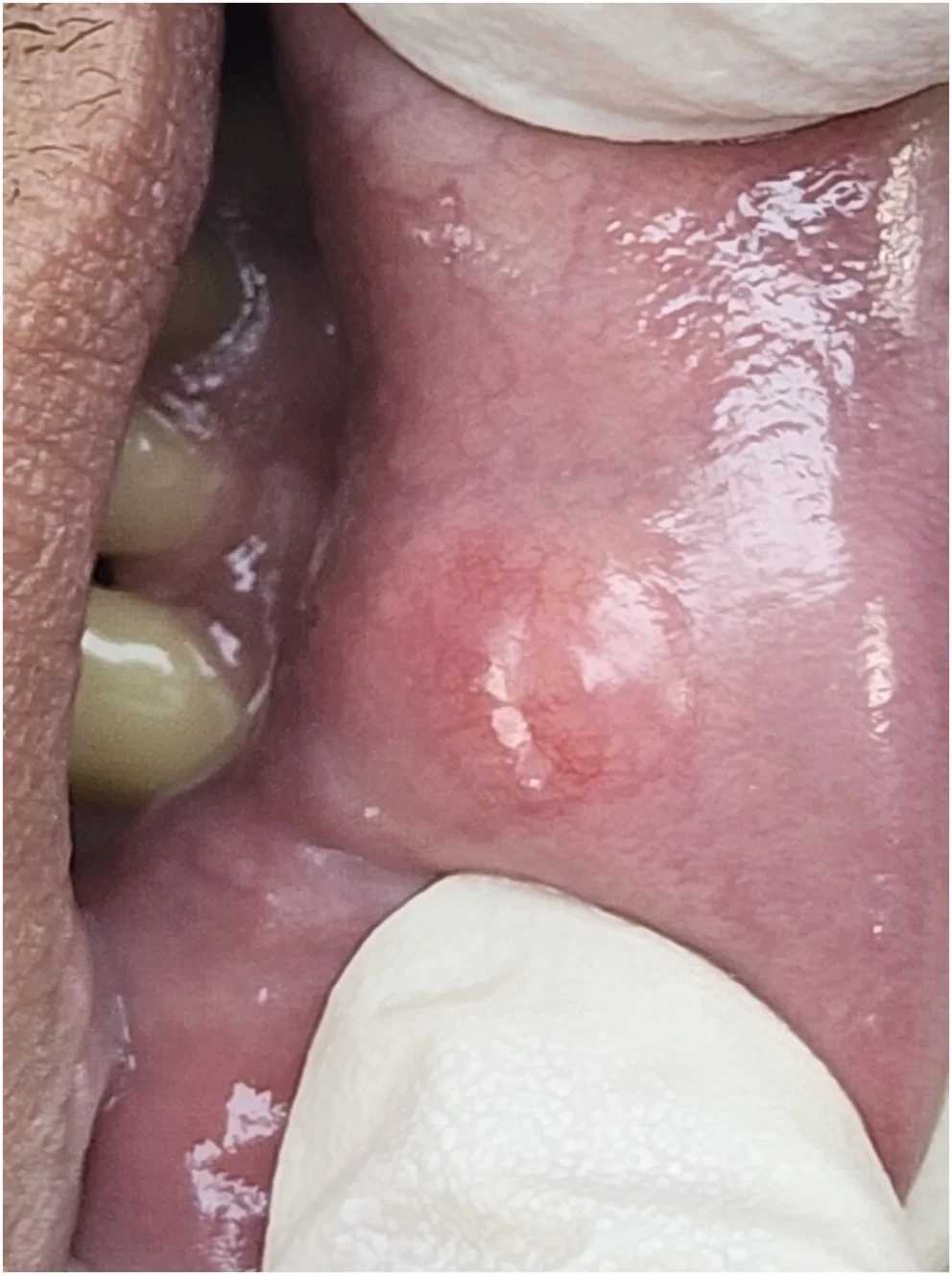
A well‐demarcated, smooth‐surfaced, pink submucosal swelling of the lower labial mucosa.

A 25‐year‐old male was referred to a private dental clinic (Tehran, Iran) with a painless submucosal swelling of the right lower labial mucosa, measuring 1 cm in diameter (Figure 1).

**FIGURE 2:**
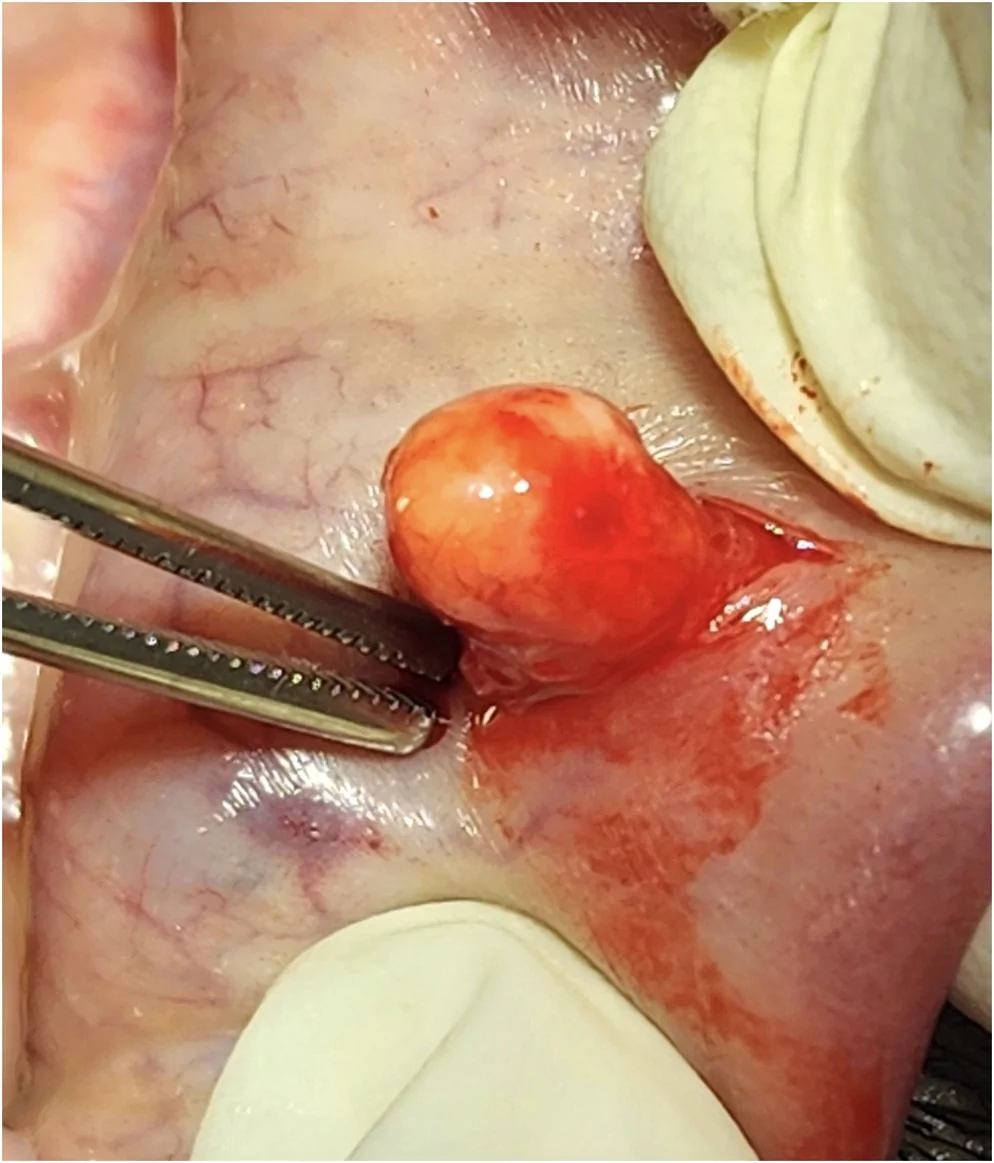
The well‐circumscribed mass is being exposed at the time of surgery.

Intraoral examination revealed a pink, circular, mobile, submucosal nodule with a firm consistency. The overlying mucosa was intact with focal hyperkeratosis. It was slow‐growing, and the patient reported that the lesion had been present for 2 years. His past medical history was unremarkable, and there was no evidence of previous trauma. The extraoral examination was normal. Finally, an excisional biopsy was performed under local anesthesia to establish a definitive diagnosis (Figure 2).

## 3 Differential Diagnosis, Investigations, and Treatment

**FIGURE 3:**
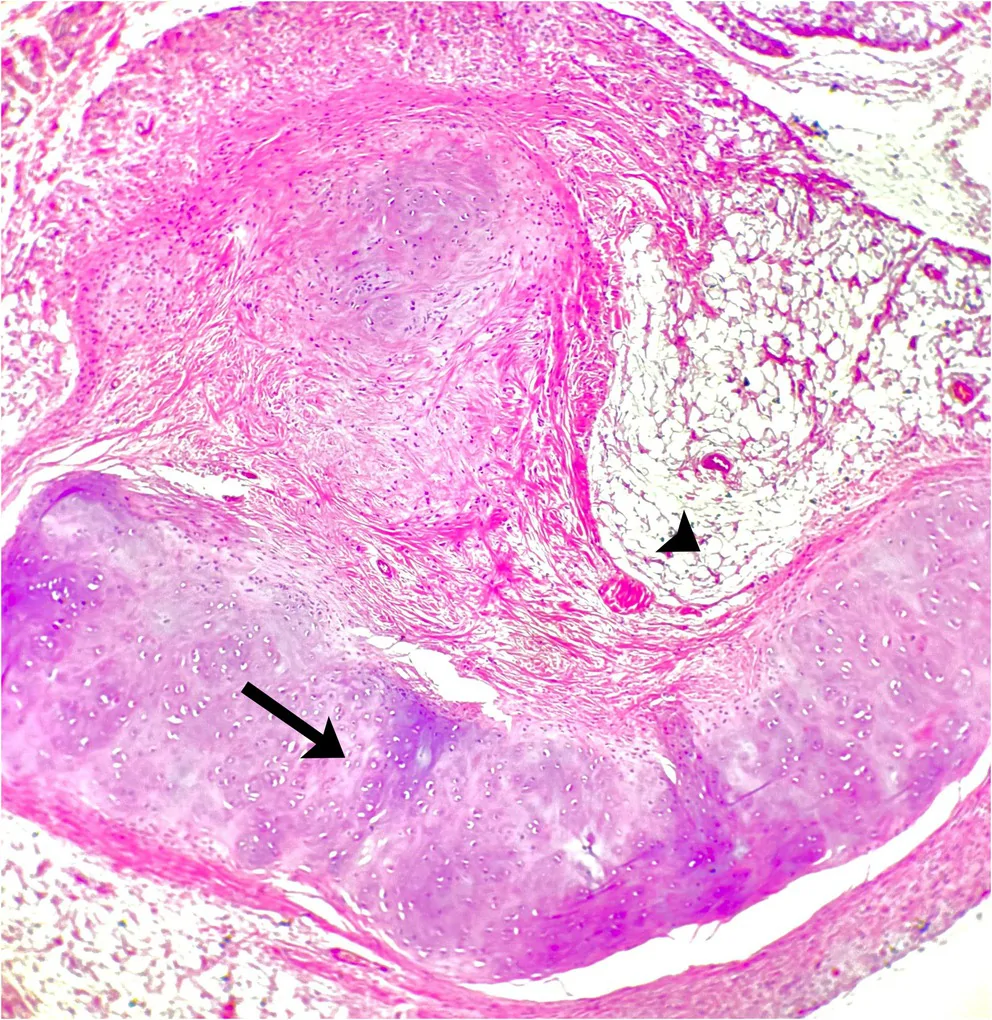
Histopathologic section displays mature cartilage (black arrow) and adipose tissue (black arrowhead) without any atypia, glandular formation, or necrosis (H&E stain, original magnification ×40).

**FIGURE 4:**
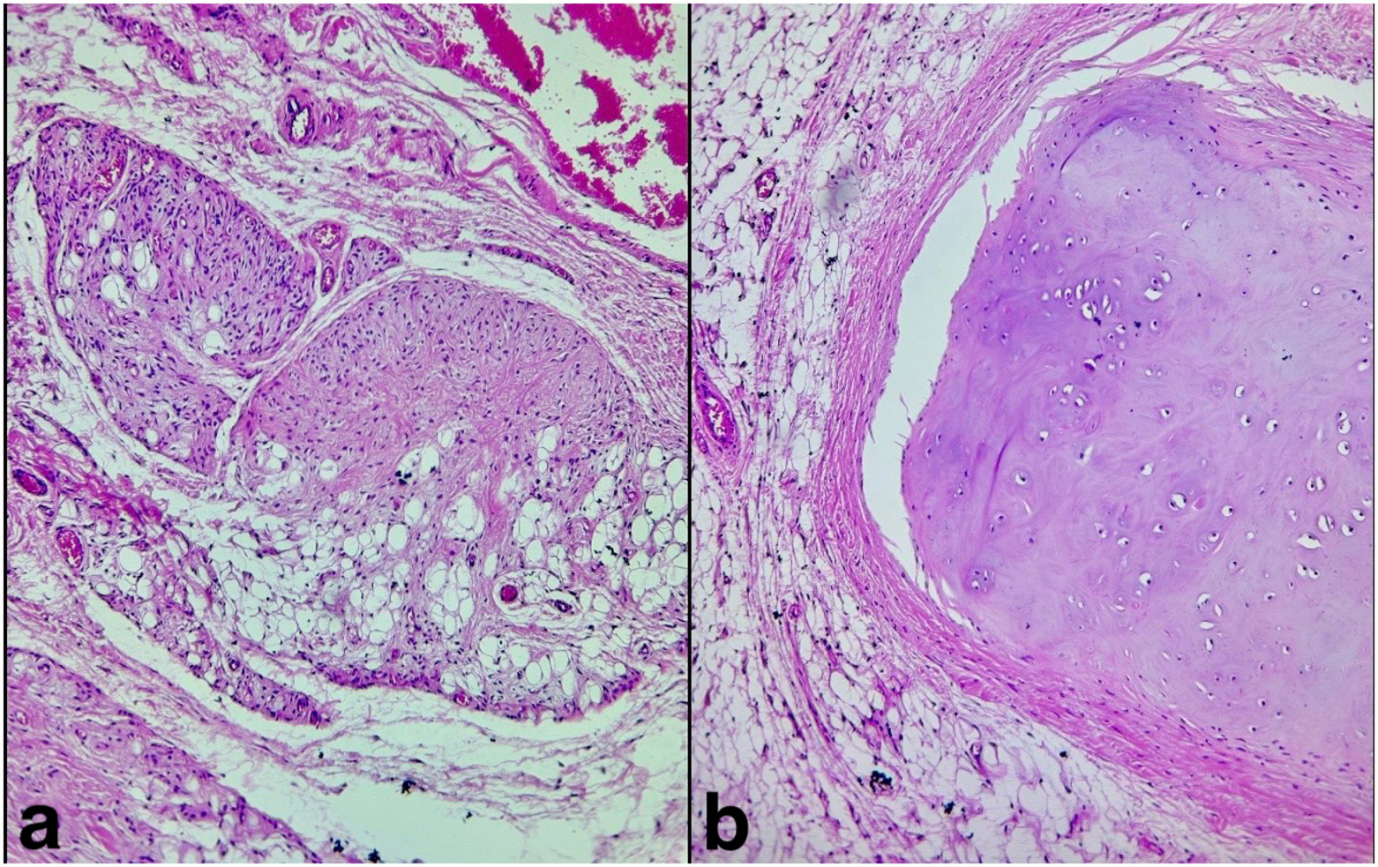
Histopathologic sections show fatty tissue admixed with bland spindle cells (a) and mature cartilage (b) (H&E stain, original magnification ×400).

Based on the lesion's location and clinical features, the differential diagnoses included mucocele and focal fibrous hyperplasia (irritation fibroma). Initial investigations, including history taking and intraoral and extraoral examinations, were nondiagnostic. In addition, the labial location of the lesion suggested a peripheral rather than central origin; combined with its benign clinical presentation, this rendered radiographic assessment unnecessary, and no radiographs were obtained. Furthermore, the biopsy specimen exhibited a solid, white, and homogeneous cut surface. Consequently, histopathologic examination with hematoxylin and eosin (H&E) staining revealed a well‐defined soft tissue lesion covered by intact oral mucosa, composed of mature cartilaginous tissue admixed with fibrous and adipose tissues with focal proliferation of bland spindle cells and no obvious cytological atypia (Figures 3 and 4). The microscopic features in combination with clinical findings were consistent with the cartilaginous choristoma diagnosis.

The biopsy was considered therapeutic, as the lesion was well circumscribed and limited in size.

## 4 Conclusions and Results (Outcome and Follow‐Up)

This case expands the limited literature on labial cartilaginous choristoma and reinforces that persistent or atypical lip nodules should undergo histopathologic evaluation. Early surgical excision provides a definitive diagnosis and appears curative. In the present case, the 6‐month follow‐up demonstrated uneventful healing of the surgical site with no evidence of recurrence.

## 5 Discussion

### 5.1 Clinicopathologic Overview

This case report describes a labial cartilaginous choristoma in a 25‐year‐old male, characterized by the presence of mature cartilaginous tissue within the oral cavity, an ectopic location for cartilage formation. However, the osseous variant represents the most prevalent type of oral choristoma, typically presenting as a firm, painless, and slow‐growing lesion located on the dorsal surface of the tongue near the foramen cecum, with a higher incidence in females during their fifth decade of life [4, 8, 10, 11, 12].

### 5.2 Literature Review

Given that the present case involves a cartilaginous choristoma of the lower lip, a literature review was performed using the PubMed and Google Scholar databases with the search terms (“lip” OR “labial” OR “oral” OR “mouth”) AND (“choristoma”) to delineate the demographic, clinical, and histopathologic features associated with this rare site of occurrence. The literature search conducted on October 7, 2025, initially identified 678 records. These records were subjected to a three‐stage screening process based on title, abstract, and full‐text review, with no restrictions applied regarding publication date. Two independent reviewers (M.K.B. and S.A.M.) performed the screening and data extraction, with disagreements resolved through consultation with a third reviewer (A.L.). Studies were excluded if they were duplicate reports, review articles, animal studies, non‐English articles without available full text, non‐labial choristomas, or reports lacking sufficient clinicopathologic information. Finally, including the present report, 13 cases of labial choristoma have been documented, with their key details summarized in Table 1.

**TABLE 1:** Demographic, clinical, and histopathologic characteristics of documented labial choristomas.

Author/Year	Age (years)	Sex	Location	Size* (CM)	Clinical findings	Clinical differential diagnosis	Histopathologic findings	Management
Pasyk et al. 1988 [13]	At birth	M	Upper Lip	3	Swelling	NA	Neuroglial	Surgery
Méndez et al. 2002 [14]	At birth	F	Upper Lip	NA	Swelling	NA	Neuroglial	Surgery
Bite et al. 1991 [15]	At birth	M	Upper Lip	1	Swelling	NA	Gastrointestinal/Respiratory	Surgery
Desmedt et al. 2007 [16]	77	F	Upper Lip	0.7	Swelling	Fibroma/Papilloma	Cartilage	Surgery
Kim et al. 2009 [5]	8	F	Lower Lip	1	Swelling	Sialolithiasis/Fibroma/Mucocele/Sialadenitis/Salivary gland tumor	Cartilage	Surgery
Saha et al. 2011 [17]	11 (Present at birth)	F	Upper Lip	2	Swelling	Fibroma/Congenital lip Pits/Congenital epulis/Dermoid Cyst/Lipoma	Cartilage	Surgery
Tariq et al. 2013 [18]	1 Month	M	Lower Lip	2.3	NA	NA	Cartilage/Osseous/Skin Adnexa	NA
Shibasaki et al. 2013 [6]	25	F	Lower Lip	2	Swelling	NA	Cartilage	Surgery
Halley et al. 2014 [8]	49	F	Lower Lip	0.5	Swelling	Sialolithiasis/Fibroma/Mucocele/Sialadenitis/Lipoma/Salivary gland tumor	Cartilage	Surgery
Bastian et al. 2015 [10]	47	F	Lower Lip	1	Pain and swelling	NA	Osseous	Surgery
Bosotti et al. 2019 [2]	57	M	Lower Lip	1.3	Swelling	Fibroma/Mucocele/Inflamed minor salivary gland/Lipoma/Minor salivary gland tumor	Cartilage	Surgery
Veni et al. 2020 [9]	43	M	Upper Lip	0.5	Pain and Swelling	Foreign body reaction	Osseous	Surgery
Present case	25	M	Lower Lip	1	Swelling	Mucocele/Fibroma	Cartilage	Surgery

Abbreviations: CM, centimeter; F, female; M, male; NA, not available.

* Note that the size of the lesion has been reported according to its greatest dimension.

#### 5.2.1 Demographic Characteristics

The demographic profile of reported labial choristoma cases revealed a wide age distribution, extending from birth to 77 years [13, 14, 15, 16], with a slight female predominance reflected in a female‐to‐male ratio of 1.17. The present case falls within this age range and represents the less frequently affected sex.

#### 5.2.2 Clinical Manifestations

In line with our case, the majority of cases were reported in the lower lip (53.85%) compared with the upper lip (46.15%). The lesion size, in its greatest dimension, ranged from 0.5 cm [8, 9] to 3 cm [13]. Most reported labial choristomas (83.33%), including the present case, presented as asymptomatic swellings. Nevertheless, evidence suggests that pain in oral choristomas can be influenced by both the lesion's anatomical location and its histopathologic type, with lingual lesions and osseous variants being more frequently associated with painful symptoms [1, 9, 10].

#### 5.2.3 Detection–Treatment Interval

The reported interval between initial patient awareness or clinical detection of the lesion and commencement of treatment varied markedly, from 5 days [14] to 11 years [17]. Pain can serve as a factor that expedites treatment, whereas asymptomatic cases often led to longer delays, unless the lesion's progressive enlargement drew the patient's concern. Moreover, routine infant examinations can facilitate early diagnosis and management.

#### 5.2.4 Differential Diagnosis and Diagnostic Challenges

Mucocele and irritation fibroma were the most frequently reported clinical differential diagnoses, consistent with the present case [2, 5, 8, 16, 17]. However, depending on the specific features of each case, other entities such as sialolithiasis, sialadenitis, papilloma, congenital epulis, congenital lip pits, dermoid cyst, lipoma, salivary gland tumor or inflammation, foreign body reactions, and other lesions may also be considered in the differential diagnosis [2, 8, 9, 17]. Additionally, the uncharacteristic clinical features of labial choristoma necessitate histopathologic investigation to establish an accurate diagnosis [8, 10]. It is noteworthy that common entities such as mucoceles or irritation fibromas, when deviating from their typical clinical course (lacking a transient nature or occurring independently of trauma), should prompt consideration of alternative diagnoses, including rare lesions such as labial choristoma. However, chronic mechanical trauma, such as habitual lip biting, may also contribute to the induction of metaplastic cartilage formation [16, 19].

#### 5.2.5 Histopathologic Classification

While oral choristomas are generally osseous in histopathologic classification [10], over half of the labial choristomas in this literature review (53.85%) were of the cartilaginous type, including the current case. Furthermore, one case presented a composite histopathology comprising cartilage, bone, and skin adnexal structures [18], and another showed a biphasic composition of gastrointestinal and respiratory tissues [15]. From a histopathologic perspective, the distinction between benign and malignant chondroid‐containing lesions, such as mixed tumors and chondrosarcomas, relies on the absence of cellular atypia, abnormal mitotic activity, necrosis, and epithelial or glandular elements within the mature cartilaginous matrix characteristic of cartilaginous choristoma [2, 5, 6, 8].

#### 5.2.6 Treatment

Local surgical excision is the preferred treatment modality, which was also employed in this report [8, 10]. To date, recurrence of oral choristoma has been reported only in the buccal soft tissue [20], whereas no recurrences have been documented in labial lesions. Nevertheless, meticulous excision remains essential due to the regenerative potential of adjacent connective tissue for chondroid formation [8, 21].

#### 5.2.7 Limitations and Future Directions

The limited number of documented labial choristoma cases constrains the depth and generalizability of the results. In addition, the follow‐up period was limited to 6 months; therefore, longer surveillance would be valuable to further confirm the absence of recurrence. Although the exact pathogenesis of this lesion remains uncertain, two principal mechanisms (embryologic and metaplastic) have been suggested to account for the presence of cartilage in oral soft tissues. The embryologic theory attributes the lesion to aberrant neural tube development, suggesting that it arises from heterotopic fetal cartilaginous rests. This theoretical framework may be particularly applicable to cases presenting “at birth” (Table 1), suggesting a congenital manifestation of the lesion. Conversely, the metaplastic theory proposes that cartilaginous choristomas originate from pluripotent mesenchymal cells, either spontaneously (de novo) or in response to triggers such as trauma or chronic irritation. This theory may provide a more plausible explanation for the underlying mechanism in adult‐onset cases of the lesion. However, lesion development may involve either a combination of the two mechanisms or the predominance of a single pathway in individual cases [2, 4, 19]. Therefore, clarifying the etiopathogenesis of oral choristomas represents an important avenue for future research.

## Author Contributions

**Kimia Hafezimotlagh:** investigation, methodology, writing – original draft. **Saede Atarbashi‐Moghadam:** data curation, investigation, project administration, writing – original draft. **Ali Lotfi:** data curation, methodology, validation. **Bahram Ardalani:** writing – review and editing. **Mohammadreza Kashefi Baher:** conceptualization, data curation, formal analysis, supervision, writing – original draft, writing – review and editing.

## Funding

The authors have nothing to report.

## Ethics Statement

The authors have nothing to report.

## Consent

Written informed consent for the publication of the patient's clinical information and associated images was obtained directly from the patient.

## Conflicts of Interest

The authors declare no conflicts of interest.

## Data Availability

All datasets generated or analyzed in this study are included in the published manuscript.
